# Readmissions – a quality metric with financial penalties?

**DOI:** 10.3402/jchimp.v3i1.20219

**Published:** 2013-04-17

**Authors:** Rohit Gulati

**Affiliations:** 1 Medical Affairs, MedStar Union Memorial Hospital, Baltimore, MD, USA; 2 University of Maryland, School of Medicine, Baltimore, MD, USA

The healthcare landscape is changing. With the current hospital payment systems, hospitals all over the nation are reimbursed for delivering care efficiently and safely across the continuum, rather than simply for each service. Incentives are provided to hospitals for improving quality and offering value to patients, both in how well they are treated and the overall patient experience. How well hospitals do in meeting these goals and achieving financial incentives relies on reducing readmissions, preventing hospital-acquired conditions, and practicing evidence-based medicine, among other pay-for-performance initiatives.

So why are readmissions so important? Besides patient safety, harm, and clinical outcomes, they are also highly cost-prohibitive. In their 2007 report to Congress: Promoting greater efficiency in Medicare, the Medicare Payment Advisory Commission stated that 17.6% of admissions result in a readmission within 30 days of discharge, accounting for $15 billion in spending. In the same report, the commission suggests that only seven conditions (heart failure, Chronic Obstructive Airways Disease (COPD), pneumonia, Acute Myocardial Infarction (AMI), Coronary Artery Bypass Graft (CABG), Percutaneous Transluminal Coronary Angioplasty (PTCA), and other vascular causes) account for over $2.2 billion in total spending for readmissions (1). Stephen Jencks in his 2010 editorial piece in the *Annals of Internal Medicine* wrote that as many as 2.5 million Medicare beneficiaries and 2 million other patients are hospitalized within 30 days of discharge, with total hospital costs (not including physician services) of about $44 billion (2). In their forecast summary for National Health Expenditure Projections 2010–2020, Centers for Medicare & Medicaid Services (CMS) states that in 2010 national health expenditure is estimated to have reached $2.6 trillion. This comes to about 17.6% of GDP. Over the projection period (2010–2020), average annual health spending growth (5.8%) is anticipated to outpace average annual growth in the overall economy by 1.1% (i.e. 4.7%). By 2020, national health spending is expected to reach $4.6 trillion and comprise 19.8% of GDP (3). It is widely acknowledged and recognized; this is not sustainable and will require the healthcare system to change in favor of value as defined by cost-effective, timely and patient-centered quality medicine rather than episodic care.

In the retrospective study by Sharma et al. (4), an analysis of the etiological factors responsible for readmissions to teaching services in a community hospital is attempted. This is the first phase of a multiphase project at a teaching hospital. They report that 63% of their study patients had three or more comorbidities, thus placing these patients at higher risk for readmissions. Multiple comorbidities are well known to be the drivers of admissions and have been validated by multiple in the US, Europe, and Australia. In a study of 29,292 patients, Silverstein (5) showed that age older than 75, male sex, African American race discharged to a skilled nursing facility with specific comorbidities predicted 30-day readmission. The comorbidities were classified by the Elixhauser (6) classification or the high-risk diagnosis in the elderly scale. What is missing from Sharma's analysis is the impact of hospital-acquired comorbidities, that is, the impact of hospital-acquired conditions on the 30-day readmission rate when all other clinical comorbidities and variables are standardized. Richard Miller (7), in a report to CMS in September 2012, suggested the impact of three common hospital-acquired conditions and their impact on the 30-day readmission rate. Miller's report suggests a strong correlation between the presence of a hospital-acquired condition and the likelihood of both readmission and discharge to a post-acute-care setting. Falls, trauma, and venous thromboembolism that were hospital acquired were associated with a 21–23% increase in the odds of being readmitted. Hospital-acquired vascular catheter-related infections had an even higher impact on readmissions. These infections were associated with a 33% increase in the odds of being readmitted within 30 days. It is becoming clear that hospital-acquired comorbidities are playing a very important part in the continuum of patient care including readmissions. Insufficient emphasis is given to hospital-acquired comorbidities or conditions. Recent data suggest that these are equally important as counting the number of comorbidities that exist at the time of admission as risk factors for readmissions. Preventing hospital-acquired conditions or comorbidities is already a quality metric but now is being linked to readmissions as well.

In their study, Sharma et al. calculated that 28% of readmissions are due to patient behavioral factors. Substance abuse is a big challenge for readmissions. These patients usually have a dual diagnosis without access to any mental health, buprenorphine, or methadone clinics. We do know that substance abuse and addiction are mental disorders and should be treated as such. This discussion on the readmissions conundrum brings back focus on the importance what this is all about: ‘Safe, cost-effective high-quality care for the entire population across the entire health care landscape’. Even if a penalty to reduce readmissions did not exist it is considered good patient care to have excellent transitions of care in place, to see if patients are taking the correct medications, adjusting to the correct lifestyle and to put in play supportive services to help patients. Some patients require more help than others. Patients who are homeless, non-compliant, and carry a dual diagnosis with substance abuse need additional help from the healthcare system. These patients are vulnerable for readmissions and also for increased mortality and morbidity. Readmissions is the first step toward true population management, and if we have to truly improve the health of the population, these special needs and possibly resource intensive patients would need that special care and follow-up. As it takes a whole village to raise a child, similarly it requires the entire community *including* the patient to raise the bar to better health. We know no single intervention implemented alone was regularly associated with a reduced risk of 30-day readmissions (8) and that is why all coordination of care measures have to be practiced uniformly in the bigger ‘system’ and across the continuum of care. We know that the standard of care for every patient with myocardial infarction is to be placed on Aspirin. Going forward, we would have to expect that the similar standards of care are uniformly applied to *all* aspects of healthcare.

Sharma also suggests that system-related modifiable causes include: medication-related problems, for example, cost, formulary coverage, prescription errors, premature discharge, poor follow-up, and so on. This has been validated by multiple studies previously. Generally, a readmission is seen as an outcome that is preceded by a number of intermediary events that, in certain circumstances, may be addressed and remedied. From August 2008 through July 2011 as part of their 9th Statement of Work (SOW), Medicare's quality improvement organizations (QIOs) in 14 states have been assessing primary factors affecting readmissions to develop interventions to target these factors (9). In their view, the causes of readmission include:

- fragmented documentation of medical conditions or failure to communicate need for medical treatment;
- poor patient self-management;
- inadequate follow-up in the post-discharge setting;
- community infrastructure and awareness problems;
- insufficient patient support, including support from family caregivers;
- medication discrepancies that occur during an initial admission or following a discharge and which may result in illness or harm to a patient.

A report from the four-state initiative to reduce readmissions states that

1. the cost of copayments for medications and follow-up visits,
2. lack of home health coverage if the beneficiary does not meet Medicare's current home-bound requirements,
3. lack of payment for transitional care services (post-discharge phone calls, coaching services, and clinical services) are factors that providers see as barriers to their efforts to reduce rehospitalizations (10).

One surprising finding in Sharma's retrospective analysis is that patients returning prematurely from nursing homes accounted for only 2.5% of the readmissions studied. Mor and colleagues in a 2010 *Health Affairs* article suggested that one-fourth of Medicare beneficiaries discharged from the hospital to a skilled nursing facility were readmitted to the hospital in 30 days, costing Medicare $4.34 billion in 2006 (11). This issue has become so important that Joseph G. Ouslander, MD, a geriatrician at the Charles E. Schmidt College of Medicine at Florida Atlantic University, is trying to improve these statistics through Interventions to Reduce Acute Care Transfers (INTERACT), a quality improvement program he developed with a colleague under a contract with the CMS.

So the big question still remains to be answered? Do readmissions define quality? Do hospitals that have high readmission rates represent poor quality of care as compared to hospitals that have lower readmission rates? Some readmissions are preventable and others are not as they just represent disease progression and complication. Can we prevent all 30 day readmissions? Definitely not. However, what we can do is come together as a healthcare system to provide the same high quality of care that is expected at all levels, inpatient and outpatient. This is the time to create a healthcare ‘system’ in which outpatient, inpatient, and transitions of care medicines are all standardized based on evidence-based medicine. All hospitals probably have protocols in reducing readmissions but have implemented them halfheartedly. In a study of 537 hospitals, Elizabeth Bradley found that even though all hospitals had written objectives of reducing preventable readmissions of patients with heart failure or AMI, the implementation of recommended practices varied widely (12). Many have said our healthcare ‘system’ is highly fragmented. A system, however, is defined as an organized set of interrelated ideas or principles. This highly organized ‘system’ is indeed coming to healthcare and will eventually define how we practice medicine. This organized healthcare system would take into account readmissions as well.

*Rohit Gulati* Medical Affairs MedStar Union Memorial Hospital Baltimore, MD, USA
University of Maryland School of Medicine Baltimore, MD, USA
